# Polybrominated Diphenyl Ethers: A Case Study for Using Biomonitoring Data to Address Risk Assessment Questions

**DOI:** 10.1289/ehp.9061

**Published:** 2006-06-12

**Authors:** Linda S. Birnbaum, Elaine A. Cohen Hubal

**Affiliations:** 1 National Health and Environmental Effects Research Laboratory, U.S. Environmental Protection Agency, Research Triangle Park, North Carolina, USA; 2 National Center for Computational Toxicology, U.S. Environmental Protection Agency, Research Triangle Park, North Carolina, USA

**Keywords:** biomonitoring, exposure assessment, PBDE

## Abstract

The use of biomonitoring data holds promise for characterizing exposure and informing risk assessment. Biomonitoring data have been used successfully to track population trends, identify susceptible populations, and provide indications of emerging environmental health issues. However, there remain challenges associated with interpreting biomonitoring data for risk assessment. An international biomonitoring workshop was convened in September 2004 to explore the use of biomonitoring data in the context of risk assessment. Six compounds were examined as case studies for this workshop, including polybrominated diphenyl ethers (PBDEs). The PBDE case study was developed to provide an example of a persistent compound for which relatively few data are available for human exposure, biomonitoring, and health outcomes. PBDEs are used in hard plastics, electronics, textiles, and polyurethane foam products. The congener pattern downstream of production facilities often resembles the commercial mixture. However, because these compounds persist in the environment and in biota, the patterns of congeners evolve. PBDEs partition into body lipids, and direct measurement of bromodiphenyl ether congeners in biologic specimens provides a good marker of exposure. Data indicate significant variability (> 100-fold range) in lipid-adjusted levels for PBDEs in the general population. It is hypothesized that both exposure and pharmacokinetics may play a role in observed congener profiles. Significant gaps in our ability to interpret PBDE biomonitoring data to address public health and risk assessment questions include limited knowledge of environmental fate and transport of PBDE congeners, limited population-based data for adults, and lack of data for potentially vulnerable populations such as children.

The use of biomonitoring data holds a great deal of promise for characterizing exposure and informing risk assessment. Biomonitoring data have been used successfully to track population trends, to identify susceptible sub-populations, to evaluate progress in reducing exposures, and to provide indications of emerging environmental health issues. However, there are significant challenges associated with interpreting biomonitoring data for risk assessment and for understanding the public health implications of measured body burdens. For example, information on exposure pathways is often required to link biomonitoring results to contaminant sources and to reduce exposures and risks. An international biomonitoring workshop was convened in September 2004 to explore the use of biomonitoring data in the context of risk assessment with an emphasis on linking measured body burdens to exposure, internal dose, and potential health outcome (Albertini et al. 2006). Six compounds were examined as case studies for this workshop: inorganic arsenic (Hughes 2006), methyl eugenol (Robison and Barr 2006), organophosphorus pesticides (Barr and Angerer 2006), perfluorooctanesulfonate (Butenhoff et al. 2006), phthalates (Calafat and McKee 2006), and polybrominated diphenyl ethers (PBDEs). These case studies were selected based on the properties of the compounds and on the richness of associated data in order to explore the potential and limitations of using biomonitoring data to inform human exposure health risk assessment. The PBDE case study was developed to provide an example of a compound that is relatively stable in the environment, with the potential to bioaccumulate but for which relatively few human exposure, biomonitoring, and health outcome data are available.

The presence and steady increase in environmental and human concentrations of PBDEs have heightened interest in the potential toxicologic consequences of these chemicals (Birnbaum and Staskal 2004; de Wit 2002). Our overall objective of this article is to discuss the use of biomonitoring data in understanding potential human health risks from exposures to PBDEs. We begin by giving a summary of the properties and uses of PBDEs. We also discuss PBDE biomarkers and the analytical methods for measuring these. Next, we highlight important data and findings on human exposure, pharmacokinetics, toxicity, and biomarker levels. Although currently available data in these areas are limited, new data are rapidly emerging. Therefore, we conclude by discussing some of the risk assessment and public health uses for these data and identifying significant gaps that should be addressed to improve interpretation of PBDE biomonitoring data.

## Properties and Uses of PBDEs

**Figure 1:**
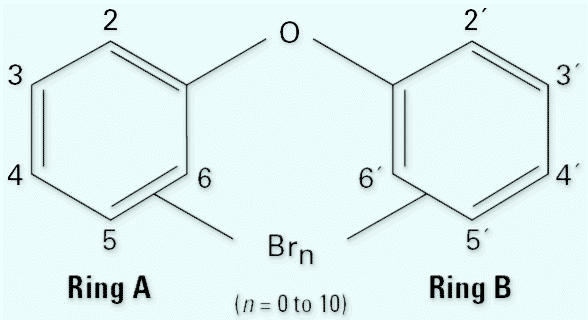
General PBDE structure.

PBDEs have the general chemical formula C_12_H(_9–0_)Br(_1–10_)O, with the sum of H and Br atoms always equal to 10. The general structure is given in Figure 1. All PBDEs present in commercial products have low water solubility (<1 μg/L) and high log octanol–water partition coefficient (*K*_ow_) values (> 5). The lower brominated congeners have higher vapor pressures than the higher brominated compounds (Birnbaum and Staskal 2004; de Wit 2002).

PBDEs theoretically comprise 209 congeners divided into 10 homolog groups (mono- to decabromodiphenyl ethers). There are three major commercial products with an average of 5 [pentabromodiphenyl ether (PeBDE), “Penta”], 8 [octabromodiphenyl ether (OBDE), “Octa”], or 10 [decabromodiphenyl ether (DBDE), “Deca”] bromines. Congeners with fewer than 4 bromines are rarely found in the commercial mixtures (Darnerud et al. 2001). PBDEs are numbered according to the International Union of Pure and Applied Chemistry (IUPAC) system originally used for polychlorinated biphenyls (PCBs). However, there are fewer congeners in the PBDE mixtures, as for polybrominated biphenyls (PBBs), than in the PCB mixtures.

PBDEs are added globally to a variety of consumer goods as flame retardants. Approximately 66,000 metric tons of PBDEs were used worldwide in 2001 [Bromine Science and Environmental Forum (BSEF) 2004]. Deca has the largest global production, with Octa being a relatively small component of the worldwide PBDE production. Penta is used primarily in North America. Although the chemical composition of the commercial Deca mixture is primarily (> 97%) DBDE (BDE-209) along with a small amount of nonabrominated diphenyl ethers (NBDEs) and OBDEs, the Octa and Penta commercial mixtures are more complex. Octa contains approximately 10–12% hexabrominated diphenyl ethers (HxBDEs), 44% heptabrominated diphenyl ethers (HpBDEs), 31–35% OBDEs, 10–11% NBDEs, and < 1% DBDE. The commercial Penta mixtures have had more variability but generally have contained 24–38% tetrabromodiphenyl ethers (TBDEs), 40–60% PeBDEs, and 4–8% HxBDEs [World Health Organization (WHO) 1994]. The major congeners in Penta are IUPAC congeners 47 and 99, which account for approximately 75% of the total mass, with approximately twice as much BDE-99 (PeBDE) as BDE-47 (TBDE). There are approximately equal amounts of BDE-153 and BDE-154 (HxBDEs), similar to that of BDE-100 (PeBDE) (WHO 1994). Octa and Penta were formally banned in Europe as of August 2004. The sole U.S. producer voluntarily agreed to stop production of Octa and Penta at the end of 2004. The structures and IUPAC numbers for common PBDEs are presented in Table 1.

The commercial mixtures have different uses, but all are additive flame retardants (BSEF 2004). Penta is primarily used in flexible polyurethane foam, where it can account for as much as 30% by weight (Hale et al. 2003). Minor uses include flame retardancy in phenolic resins, polyesters, and epoxy resins. Octa is used as an additive in polymers for use in plastic housing and office equipment. Deca is used in high-impact polystyrene and as a polymer backcoating on commercial textiles and is added to a wide variety of polymers used in electrical and electronic equipment.

## Biomarker of Exposure

Several studies have demonstrated that the PBDEs partition into body lipids. Similar results are obtained from blood, serum, cord blood, breast milk, and adipose tissue if measurements are normalized to lipid (Hites 2004). There are no reports of PBDE measurements in urine.

Because of their persistence and bioaccumulation, measurement of BDE congeners in biologic specimens is a good marker of exposure. To date, little attention has focused on measuring potential BDE metabolites as biomarkers.

Measurement of PBDE congeners usually requires high-resolution gas chromatography/mass spectrometry techniques. Under most measurement conditions, most congeners can be detected below the parts-per-billion range. However, because of the ubiquitous presence of Deca, there are problems with laboratory contamination with BDE-209, and special care must be taken. In addition, because there are so many potential congeners, it is essential that standards be used and the identity of specific congeners confirmed. For human samples, values are usually reported on a lipid-adjusted basis. This allows comparison across various matrices, such as blood, breast milk, and adipose tissue. This is not always done for animal samples, where wet weight is often used. Often to estimate intake from food, it is more important to know the wet weight.

## Exposure Assessment

Until very recently, data were not available to attempt a mass-balance comparison of measured body burden levels with exposure estimated indirectly from information on product use, measurements in environmental media, and human activity and pharmacokinetics. Such an analysis would still be difficult because of limited availability of data, variability in body burdens, and lack of information on the fate and transport of the commercial products and the PBDE congeners.

Recent studies have demonstrated that PBDE congeners have been detected in all people measured in North America, at levels of 5–10,000 ng/g lipid. The average levels are approximately 10 times those in Europe or Asia, where the range of measured PBDE congeners is 1–10 ng/g lipid. Based on the available data, there appears to be much greater variability (approximately a 100-fold range) in the lipid-adjusted levels than observed for other persistent organic pollutants. For example, although 5% of the general population has lipid-adjusted concentrations of dioxins and PCBs that are double the mean, and 1% has concentrations that are triple, 5% of the population has concentrations of PBDEs at least 10 times the average (Schecter et al. 2003).

The wide variation in human levels introduces some questions about exposure assessment using biomarkers. Although BDE-47, the major congener found in people, is very persistent in the rat, it is rapidly eliminated unchanged in the urine of mice (Orn and Klasson-Wehler 1998). Recent studies from Staskal et al. (2005) have suggested that BDE-47 may be a substrate for an active transport system in the kidney and/or may be highly bound to urinary proteins.

The PBDEs are always present as mixtures. Because there is growing evidence for the ability of both photolytic (Soderstrom et al. 2004) and biotic (Stapleton et al. 2004) debromination to occur, it is impossible to determine for sure which PBDE product is responsible for the general population exposure. In addition, many published studies have measured only a very limited number of congeners, and the choice has not been consistent. Butt et al. (2004) have shown that BDE-209 is the major PBDE in air and on window films; previously, it was not often measured. Similarly, some human studies have examined only BDE-47 (e.g., Petreas et al. 2002) or a limited number, including BDE-99, BDE-100, BDE-153, and BDE-154 (e.g., Mazdai et al. 2003), with no measures of higher brominated species that could come from Octa or Deca mixtures.

The congener pattern downstream of production and/or use facilities often resembles the commercial mixture (Hale et al. 2001, 2003). However, because these compounds persist and bioaccumulate up the food chain, the patterns of congeners evolve (Alaee et al. 2002). Although the ratio of BDE-99 to BDE-47 is nearly 2 in the commercial Penta product, in most human samples, this ratio is inverted (e.g., Schecter et al. 2003). In some marine mammals high on the food chain such as ring seals in northern Canada, the levels of BDE-99 are barely 1/10th of those of BDE-47 (Ikonomou et al. 2002). This is likely due to metabolism of BDE-99 because it has been shown to be well absorbed and converted to hydroxy and debrominated hydroxy metabolites (Hakk et al. 2002). In addition, the relative amounts of several other penta and hexa congeners change. Whether these changes are due to metabolism and clearance or are the result of debromination from the Octa and Deca mixtures remains to be determined.

Indirect estimates of exposure have been conducted by industry under the Voluntary Children’s Chemical Evaluation Program for the U.S. Environmental Protection Agency (Toxicology Excellence for Risk Assessment 2003a, 2003b). These estimates focused on exposure to children but did include some occupational estimates. Unfortunately, these exposure estimates were based on European air, soil, and food levels, which are much lower than those observed in the United States.

There is very limited information concerning the source–exposure–dose continuum. Little quantitative information is known regarding how the PBDEs are entering the environment, although hypotheses include leakage at site of use and incorporation into products, during use of the products, and during destruction. Exposures have been studied for workers involved in manufacturing PBDEs or handling products containing flame retardants (Sjodin et al. 1999, 2001; Thuresson et al. 2005). PBDEs can be found in sediment downstream of use sites (Hale et al. 2003). PBDEs have been measured in air, at higher concentrations in rooms with electronics equipment, and in dust where equipment as well as furniture and heavy textiles are present. Recently, several studies have measured PBDE concentrations in various foods (Harrad et al. 2004; Huwe et al. 2002b; Schecter et al. 2004), including fish (Hites et al. 2004). In the United Kingdom (Harrad et al. 2004), estimates of daily intake are 2 ng/kg/day, similar to what has been estimated in Spain (Bocio et al. 2003). Schecter et al. (2004) published the first preliminary study of supermarket food in the United States. Calculations based on this information suggest that daily intake varies by age. Nursing infants may be exposed to 150 ng/kg/day due to lactational off-loading, whereas most adults would consume approximately 1–2 ng/kg/day “total” PBDEs (sum of 13 congeners). Wilford et al. (2004) measured the lower brominated PBDE congeners in indoor and outdoor air in Canada. On the basis of median PBDE levels found in this study, the authors estimate that approximately 4% of overall daily intake is the result of inhalation exposures. These limited data suggest that foods of animal origin are a significant source of human exposure, but inhalation and dust may also be important. In fact, a recent report from Webster et al. (2005) demonstrates a clear association between the sum of the five major lower brominated congeners in breast milk and those in house dust.

## Pharmacokinetics

### General information

PBDEs can break down at high temperatures. These also undergo ultraviolet (UV)-catalyzed debromination (Soderstrom et al. 2004). Although earlier studies had indicated that the rate of debromination was inversely related to the degree of bromination, with DBDE breaking down most rapidly, at least in organic solvents, recent studies have demonstrated that this breakdown also occurs in sand and sediment. BDE-47 could be produced in the presence of organic solvents from BDE-209 with UV light, as could multiple penta, hexa, hepta, octa, and nona congeners (Soderstrom et al. 2004). Anaerobic microbial degradation of Deca also appears to occur, although relatively slowly (Gerecke et al. 2005). The congener composition found in biota rarely mimics that found in the commercial mixtures: not only are congeners detected that have not been reported in the commercial mixtures, but the ratios of congeners change (Hites 2004). For example, although the ratio of BDE-99 to BDE-47 is nearly 2 in the commercial Penta mixture, there is almost twice as much BDE-47 as BDE-99 in human breast milk (Schecter et al. 2003).

### Penta-specific information

A commercial Penta product was given orally to rats, and the half-life of individual congeners was determined in adipose tissue (Von Meyerink et al. 1990). Half-lives were 19–30 days for TBDE, 42–25 days for PeBDEs, and 50–105 days for HxBDEs.

To date, there have been two toxicokinetic studies published for BDE-99 in rats (Chen et al. 2006; Hakk et al. 2002), demonstrating that this congener is well absorbed, distributed to lipid-rich tissues, and metabolically eliminated. Orn and Klasson-Wehler (1998) studied the fate of BDE-47 in both mice and rats after a single oral dose. Although its behavior was as expected in rats (well absorbed, distribution to adipose and liver, long half-life, elimination in feces, limited metabolism), the parent compound was rapidly eliminated in the urine of mice. These results have been confirmed by Staskal et al. (2005) in another strain of mouse and by Sanders et al. (2006) in rats. Staskal and co-workers have also demonstrated that the pharmacokinetic behavior of BDE-47 is dose dependent, with relative elimination decreasing as the dose is raised. They have shown that BDE-47 is well absorbed after oral, intratracheal, and intraperitoneal exposure (> 80%) and that dermal absorption is > 60%. Less than 5% of an administered dose remains in the mice 21 days after treatment, but the terminal half-lives are longer than 20 days, suggesting some potential for bioaccumulation. Elimination is consistently greater in urine than in feces and is primarily protein-bound unmetabolized BDE-47 (Staskal DF, Birnbaum LS, personal communication). Pharmacokinetic behavior changes as a function of age that could explain the increased susceptibility to developing animals (Eriksson et al. 2002).

### Octa-specific information

Limited studies have been conducted on the commercial Octa mixture (Huwe et al. 2002a).

### Deca-specific information

Several studies have suggested that BDE-209 is poorly absorbed, both from the gastrointestinal tract [National Toxicology Program (NTP) 1986] and from the skin (Hughes et al. 2001). However, recent studies have indicated that DBDE can be absorbed by the rat with bioavailability of approximately 26% when a suitable vehicle was used (Morck et al. 2003). Approximately 10% of the administered dose was eliminated as metabolites via the bile. Hydroxylated NBDEs and OBDEs were produced, demonstrating debromination. The terminal half-life was surprisingly short: 2.5 days. This may be a reflection that peak concentrations were not reached in the rat during the course of this short study because there was no evidence of high concentrations of BDE-209 in adipose tissue, which is normally a major depot for such a lipophilic compound. In fact, in a long-term feeding study in rats using the commercial Deca mixture, retention was observed in fat (Kociba et al. 1975). Once again, uptake and retention may vary as a function of age. In newborn mice the uptake of DBDE is as high as for PeBDE (Viberg et al. 2003b).

### Human data

Recently, Geyer et al. (2004) estimated terminal elimination half-lives in humans for BDE-47, BDE-99, BDE-100, BDE-153, and BDE-154. The half-lives estimated from body burden and daily intake (based on market basket survey in Sweden) were on the order of 2–3 years, with the exception of BDE-153 for which estimated half-lives were on the order of 4–6 years or more. These estimates assumed gastrointestinal absorption from food of 86–96%. Estimates of half-lives in human fat extrapolated from measured half-lives in the fat of rats were significantly greater. Higher brominated congeners appear to have shorter half-lives. Thuresson et al. (2006) reported apparent half-lives in human serum of 11–18, 18–39, and 37–91 days for deca, nona, and octa BDEs, respectively.

## Toxicity Data

### Animal toxicity

#### General information

Limited studies have been conducted concerning the ecologic effects of PBDE (Birnbaum and Staskal 2004; Birnbaum et al. 2003). None of the commercial mixtures are genotoxic. In general, Penta is more toxic than Octa, whereas Deca is essentially nontoxic to invertebrates. Recent studies have shown Penta to be developmentally toxic to fish (Timme-Laragy et al. 2006). Mammalian toxicity studies have been conducted on all three commercial mixtures, and in general, relatively high doses are required for overt toxicity in adults. However, there is growing concern for developmental and endocrine effects. Limited information is available on congener-specific effects. A growing concern is their structural similarity to other polyhalogenated aromatic hydrocarbons, such as PCBs and dioxins (Darnerud et al. 2001). The commercial mixtures contain dioxin-like contaminants (Sanders et al. 2005), which may also be generated upon burning. Although some PBDE congeners may have weak affinity for the aryl hydrocarbon receptor, the congeners that are found in environmental samples and biota are not dioxin-like (Chen et al. 2001).

#### Penta-specific information

Penta has been implicated in hepatic and endocrine-related effects after repeated exposure but has relatively low acute toxicity. The commercial Penta can induce both phase I and phase II hepatic enzymes. Penta exposure leads to a decrease in serum thyroxine levels (Zhou et al. 2002), as does exposure to BDE-47 or BDE-99. In fact, decreases in thyroxine have been reported in mice after a single dose of 0.8 mg Penta/kg (Fowles et al. 1994). Several mechanisms have been proposed for these effects, including induction of hepatic glucuronyl transferases (Zhou et al. 2001) and binding to transthyretin (Meerts et al. 2001). There are some reports of immunotoxicity from Penta, but this is likely due to dioxin-like contaminants in the mixture.

Developmental reproductive effects occur after maternal exposure. These include delays in puberty and decreases in sex organ weights. Recent reports have indicated that Penta, as well as the major PBDE congeners that compose it, may be antiandrogenic (Stoker et al. 2004, 2005). Other experimental work has suggested that certain PBDEs or their metabolites may be estrogenic or antiestrogenic (Lilienthal et al. 2006; Meerts et al. 2001).

The greatest toxicity concern is the potential for Penta as a developmental neurotoxicant. A handful of studies have demonstrated alterations in both rats and mice. An extensive series of studies from the laboratory of P. Eriksson at Uppsala University in Sweden (Eriksson et al. 2001; Viberg et al. 2002, 2003a, 2003b, 2006) has shown that BDE-47, BDE-99, BDE-153, and BDE-209 impair spontaneous motor activity, impair cholinergic transmitters, and disrupt habituation. These results suggest negative effects on learning and memory that worsen with age. This group of researchers also defined a critical window of susceptibility that occurs during a period of rapid brain growth: when exposed on postnatal day 10, the aforementioned effects ensue, most of which are not reversible and often worsen with age. Specifically, BDE-99 has been shown to be developmentally neurotoxic in at least three strains of mice and in both genders. Branchi et al. (2003), Kuriyama et al. (2005), and Talsness et al. (2005) also saw effects of BDE-99 after developmental exposure of mice and rats. Developmental neurotoxicity has also been reported in rats, but relatively high gestational and lactational exposures to commercial Penta were required. This is reminiscent of the situation with PCBs where developmental neurotoxicity is observed at lower doses in mice than rats. Penta has also been shown to affect neuronal cell signaling *in vitro* (Kodavanti and Derr-Yellin 2002; Mariussen and Fonnum 2003).

There are no chronic studies of Penta. However, the NTP has plans to conduct 2-year studies of both the commercial mixture and the three individual congeners, BDE-47, BDE-99, and BDE-153 (NTP 2006).

#### Octa-specific information

Octa has low acute toxicity; effects are generally observed in liver and endocrine tissue after exposure. In a single developmental study, Octa led to fetal toxicity in the rat at doses below those at which effects were seen in the dams (WHO 1994). Effects were observed in developing rabbits at a dose of 2 mg/kg to the mother. BDE-153, a congener present in Octa, has been shown to be developmentally neurotoxic (Viberg et al. 2003a). No chronic studies have been reported on Octa.

#### Deca-specific information

The NTP (1986) has conducted extensive acute and chronic studies on Deca in both rats and mice. High doses (up to 50,000 ppm in the diet) resulted in liver tumors in rats and male mice. There was some indication of an increase in thyroid gland tumors. Deca had few other effects (Darnerud 2003). However, BDE-209 has been shown to be developmentally neurotoxic (Viberg et al. 2003b).

### Human toxicity

There are almost no data on the toxicity of PBDEs in humans. There is no evidence for Deca causing skin sensitization (WHO 1994). There was some evidence of primary hypothyroidism and reduction in nerve conduction velocities after occupational exposure to both PBDEs and PBBs (Bahn et al. 1980). In male fish eaters from the Baltic, Hagmar et al. () concluded that their results did not support a role in adult men for dietary exposure to persistent organochlorines, including BDE-47, and effects on peripheral hormone levels, including thyroid, were not observed. A small study (*n* = 12) also failed to show any association between PBDE levels and thyroid hormones (Mazdai et al. 2003).

## Environmental Public Health Use of Biomonitoring Data

Currently, our ability to use PBDE biomonitoring data to address risk assessment and public health questions varies based on the particular questions we are trying to answer. Because the BDEs congeners can be measured directly in biologic tissues, biomonitoring has been used effectively to measure human body burdens and to identify exposure to PBDE as a potentially important environmental health issue.

Characterizing the exposures that are resulting in measured body burdens has been more difficult. Because PBDEs occur as mixtures, understanding the changes in congener ratios from sources to exposure media to internal dose (i.e., the fate and transport of PBDEs in the environment) is required to assess exposure. Currently, little is known about the sources of human exposure and how PBDEs are entering the environment. Additional environmental monitoring data (particularly data collected concurrently with biomonitoring data) would help to identify sources of exposure to better focus future environmental health studies.

On the other hand, biomonitoring of PBDEs has been effective for demonstrating potential trends in exposure. The levels of PBDEs have increased exponentially since the early 1970s. Although this trend peaked in Sweden in the late 1999s (Meironyte et al. 2001), levels continue to increase in North America. Recent studies have demonstrated that the median body burdens, based on lipid adjustment, are approximately 10 times higher in the United States than in Europe (Hites 2004; Sjodin et al. 2004). People with the lowest concentrations in the United States are more highly exposed (internally) than people with the highest exposure levels in Europe today. These data can also be used to characterize population variability. In both the North American and European populations there is a wide range of body burdens. Unlike PCBs and dioxins, approximately 5% of the population has levels 10 times the mean PBDE concentration, and 1% are as much as 50 times the mean (Environmental Working Group 2003). Whether this unusually broad range is due to differential exposure within the general population or to biologic mechanisms remains to be determined. In addition, very limited European data indicate that young children have significantly higher BDE levels than do adults. Thus, more extensive biomonitoring at the population level is required to determine reference levels and to characterize vulnerable populations such as children. The lack of biomonitoring data in children is likely related to the current requirement for blood or adipose tissue to measure BDE congeners. Development of PBDE biomarkers in urine would facilitate study of children.

In addition to being used to characterize exposure, biomonitoring data are often used to link exposure with potential adverse health outcomes. Given the understanding that internal dose is the best metric for relating dose to response, the body burden measurements from biomonitoring offer the most appropriate approach for relating the levels in humans to those in experimental animals in which effects have been observed. This approach is most effective for persistent, bioaccumulating toxicants such as the PBDEs.

Multiple health effects have been reported in experimental animals after exposure to commercial PBDE mixtures, as well as exposure to several of the most important congeners. However, which effect and which study to use as the point of departure for risk assessment remain to be determined. In general, developmental neurotoxicity appears to be an extremely sensitive end point, at least in mice. Recent studies suggest that developmental reproductive effects may have similar sensitivity, as do effects on thyroid hormones. In mice, these effects have been observed after a single dose of < 1–10 mg/kg. Assuming that absorption is > 80%, this would lead to a body burden at the critical window of < 0.8–8 mg/kg. The most highly exposed person to date has a body burden of approximately 1.5 ng/g lipid, equivalent to approximately 0.3 ng/g or 0.3 mg/kg. Comparison of the body burdens in highly exposed people with those in affected mice suggests that the margin of exposure could be < 10.

Finally, there have been no large-scale epidemiology studies for PBDEs and effects in humans are unknown. To better understand the potential public health implications of available PBDE biomonitoring data, additional study is required. Significant insight could be obtained from additional information on environmental fate and transport of PBDE congeners, as well as population-based biomonitoring data for adults and for potentially vulnerable populations such as children.

## Figures and Tables

**Table 1:** Most often measured PBDE: IUPAC numbers and structures.

IUPAC number	Bromine substitution structure
17	2,2′,4
28	2,4,4′
47	2,2′,4,4′
66	2,3′,4,4′
77	3,3′,4,4′
85	2,2′,3,4,4′
99	2,2′,4,4′,5
100	2,2′,4,4′,6
138	2,2′,3,4,4′,5′
153	2,2′,4,4′,5,5′
154	2,2′,4,4′,5,6′
183	2,2′,3,4,4′,5′,6
209	2,2′,3,3′,4,4′,5,5′,6,6′
